# Piperazine-Substituted Pyranopyridines Exhibit Antiproliferative Activity and Act as Inhibitors of HBV Virion Production

**DOI:** 10.3390/ijms26093991

**Published:** 2025-04-23

**Authors:** Sona Buloyan, Arpine Harutyunyan, Hrachik Gasparyan, Anahit Sakeyan, Astghik Shahkhatuni, Natalia F. Zakirova, Gaukhar Yusubalieva, Ilya M. Kirillov, Irina T. Fedyakina, Pavel N. Solyev, Anastasia V. Lipatova, Mikhail A. Bogomolov, Vladimir S. Prassolov, Timofey D. Lebedev, Alexander V. Ivanov

**Affiliations:** 1 Scientific Technological Center of Organic and Pharmaceutical Chemistry, National Academy of Sciences of the Republic of Armenia, 0019 Yerevan, Armenia; sonabuloyan@gmail.com (S.B.);; 2 Engelhardt Institute of Molecular Biology, Russian Academy of Sciences, 119991 Moscow, Russia; 3 Federal Research and Clinical Center of Specialized Medical Care and Medical Technologies, FMBA of Russia, 115682 Moscow, Russia; 4 Gamaleya National Research Centre for Epidemiology and Microbiology of the Ministry of Russia, 123098 Moscow, Russia

**Keywords:** pyranopyridine, piperazine, antiproliferative activity, apoptosis, hepatitis B virus, virion production

## Abstract

Advances in medicinal chemistry have led to the development of anticancer and anti-infectious drugs. However, many types of cancer and viral infections such as hepatitis B virus or SARS-CoV-2 are still treated ineffectively. Therefore, further development of effective and selective lead compounds as potential drugs is still highly demanded. In this study, we synthesized a novel series of piperazine-substituted pyranopyridines and evaluated their anticancer and antiviral properties. Antiproliferative activity was determined in a panel of various tumor cell lines as well as non-tumor hepatic HepaRG cells. Mechanisms of cytotoxicity were assessed by fluorescent microscopy techniques. Antiviral activity was analyzed towards DNA and RNA viruses in infectious cell culture systems. Several compounds showed antiproliferative activity towards various cancer cell lines at micromolar and submicromolar concentrations. Mechanisms of cytotoxicity involve the induction of apoptosis and are not mediated via ERK1/2 pathway or oxidative stress. Several compounds exhibit selective activity against hepatitis B virus by preventing the formation of virion particles. This study led to the identification of a novel class of piperazine-substituted pyranopyridines with antiproliferative activity towards a wide range of tumor cell lines as well as the non-toxic inhibitor of HBV virion production.

## 1. Introduction

Cancer poses a significant social, public health, and economic challenge, accounting for nearly one in six deaths globally and, in particular, one in four deaths from non-communicable diseases [1]. The most commonly diagnosed cancers worldwide include lung, colorectal, and prostate cancers, which continue to be the leading causes of cancer-related mortality [2]. Despite the therapeutic advances that have improved progression-free and overall survival rates, more endeavors are required to make cancer curable [3]. Unfortunately, cancer treatments often lead to side effects and impairments that are greatly feared by both patients and clinicians. It is crucial to identify, manage, and prevent these adverse effects [4]. Additionally, cancer heterogeneity, evolution, and the influence of local and systemic environments play pivotal roles in disease development, therapeutic response, resistance, and recurrence [5]. The most effective strategy for continually reducing global cancer mortality involves the widespread implementation of precise and individualized treatment and increased investment in advancing cancer drug research [6].

Outbreaks of novel virus infections together with the wide spread of many other viral pathogens represent another challenge for public health, as demonstrated by the COVID-19 pandemic, which has claimed over 6 million lives [7]. The pandemic highlighted the urgent need for effective antiviral agents. However, despite recent advances in antiviral drug development, viruses mutate rapidly, significantly diminishing the therapeutic efficacy of existing medications. Consequently, addressing emerging viral threats and combating drug resistance remain critical goals in antiviral drug discovery [8]. Direct-acting antivirals are required to decrease burden of chronic infections including human immunodeficiency virus (HIV) and oncogenic hepatitis viruses B, C, and delta [9,10].

The utilization of hybrid molecules with a dual mode of action to create efficient new drugs is very promising as distinct features of each component can be hybridized and their properties leveraged [11,12,13]. In this regard, a combination of two heterocyclic compounds, piperazine and pyridine, can be prominent for the synthesis of new bioactive drugs.

Piperazine is a structural element that is present in various chemical classes and is used for numerous different therapeutic applications. This structure has been considered a privileged scaffold for drug design [14]. Multiple studies of the biological activities of piperazine derivatives showed that they possess many pharmacological properties. For instance, azole-containing piperazine derivatives showed antibacterial and antifungal activities [15,16], diphenylalkyl piperazine derivatives exhibit antioxidant activity [17], and piperazine ferulate exerts an antihypertensive effect and improves endothelial function [18]. Piperazine is considered a key substructure for the development of new antidepressants [19]. Several derivatives of piperazine also demonstrate antiviral [20], anticonvulsant [21], and acetylcholinesterase inhibition effects [22]. Recently, piperazine-containing compounds were reported as promising agents for anticancer therapy as they exhibit an antiproliferative effect on various human cancer cell lines [23,24].

Pyridine is considered another interesting chemical scaffold that is incorporated into the structures of many pharmaceuticals [25]. Various derivatives of pyridine may also act as antimicrobial [26], antiviral [27], antioxidant [28], anticancer [29,30], antidiabetic [31], antimalarial, analgesic, or anti-inflammatory agents [32].

**Scheme 1:**
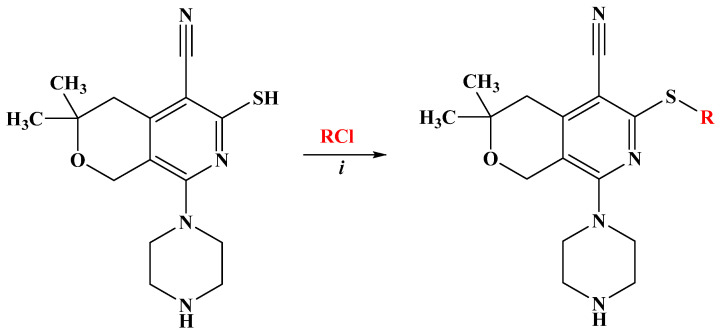
Synthesis of pyrano[3,4-*c*]pyridine derivatives **D011-37**, **D011-42**, and **D011-45**–**D011-50**. (*i*) Na_2_CO_3_, H_2_O, C_2_H_5_OH, 6 h, and r.t.

Taking into account these facts, we designed and synthesized new derivatives incorporating two pharmacophore moieties, piperazine and pyridine, as a hybrid molecule [33]. In the present study, the synthesized hybrid molecules (3,3-dimethyl-8-piperazin-1-yl-3,4-dihydro-1H-pyrano[3,4-c]pyridines) have been analyzed for their anticancer, antioxidant, and antiviral properties to identify either non-toxic compounds with antiviral activity or compounds with pronounced antitumor activity. They comprised a piperazine-substituted pyranopyridine backbone (black) and a thioacetamide (**DO11-37**) or thioaryl side group (red) with substitutions in various positions of the phenyl ring (**DO11-42**, **DO11-45**–**DO11-50**) (Scheme 1).

## 2. Results

### 2.1. Chemistry

Based on the fact that functionally substituted piperazines possess a broad spectrum of biological activity and have found their application in medicine [34,35], we have developed a method for obtaining thioalkyl derivatives of piperazine-substituted pyrano[3,4-c]pyridines. The synthesis of compounds **D011-37**, **D011-42**, **D011-45**, **D011-46**, **D011-47**, **D011-48**, **D011-49**, and **D011-50** is outlined in Scheme 1.

As the starting compound, 8-piperazin-1-yl-5-cyano-6-thio-pyrano[3,4-c]pyridine [36], which has three reaction centers, was used. While electrophilic substitution is possible in the piperazine ring, in the thio group, and at the endocyclic nitrogen atom, we have developed suitable conditions that allow regioselective substitution specifically at the thio group of the pyridine ring, leading to the preparation of new *S*-substituted compounds (**D011-37**, **D011-42**, **D011-45**, **D011-46**, **D011-47**, **D011-48**, **D011-49**, and **D011-50**) (Table 1).

The structures of newly synthesized compounds were confirmed by IR and NMR spectroscopy, HRMS, and elemental analysis (see Supplementary Materials).

### 2.2. Piperazine-Substituted Pyranopyridines Display Antiproliferative Activity

Our initial goal was to investigate possible antiproliferative activity of the compounds towards a panel of various tumor cell lines. This panel included lung (A549), cervix (HeLa), prostate (DU145) carcinomas, chronic myeloid leukemia (K562), and neuroblastoma (SH-SY5Y), as well as two low-passage glioblastoma (GBM5522 and GBM6138) cell lines. As a control, we used the liver progenitor HepaRG cell line differentiated into a mixed population of hepatocyte- and cholangiocyte-like cells [37]. It is noteworthy that HepaRG cells are negative for liver cancer markers such as α-fetoprotein and cannot proliferate indefinitely [38]. This cell line is widely used by toxicology as it expresses high levels of cytochromes P450 that are involved in metabolism of xenobiotics [39]. Antiproliferative activity was analyzed using a widely used MTT assay. As controls, we also tested two anticancer drugs (5-fluorouracil and sorafenib) with different mechanisms of action and thus with different levels of antiproliferative activity.

All compounds not only decreased cell proliferation but triggered cell death. The least toxic compound was **DO11-37**, while the most toxic compounds were **DO11-42**, **DO11-46**, **DO11-48,** and **DO11-49** (Table 2). As their IC_50_ values were below 10 µM, they meet the requirements of National Cancer Institute (NCI) for initial selection of compounds for subsequent evaluation as anticancer agents [40]. Prostate carcinoma DU145, cervix carcinoma HeLa, and leukemia K562 cell lines were the most sensitive to these compounds, with the best IC_50_ values being submicromolar. Lung carcinoma A549 and glioblastoma GBM5522 cells were a bit less sensitive to the drugs, though the best IC50 values were also in a low micromolar range. Most compounds were much less sensitive towards the other glioblastoma cell line (GBM6138), though **DO11-49** had equal toxicity towards both glioblastoma cell lines.

The highest selectivity indexes were demonstrated in the case of DU145 cells by **DO11-46** (SI 38) and **DO11-48** (SI 48). This indicates that high antiproliferative activity requires either a chlorophenyl-substituted motif or 2-methoxy sterically hindered fragment. This configuration of substituents may form a coordinating sphere with several heteroatoms (-S-, -NH-, -OCH_3_, or -Cl, -CN) aligned around it. Considering chlorine’s ability to form halogen bonding, it can be assumed that the electron donating nature of the substituents in the phenolic ring enhances the antiproliferative activity. Finally, **DO11-37** and **DO11-47** were almost non-toxic for non-cancer HepaRG cells, indicating that they could be evaluated for other types of biological activity.

### 2.3. The Piperazine-Substituted Pyranopyridines Induce Apoptosis and Necrosis

**Figure 1:**
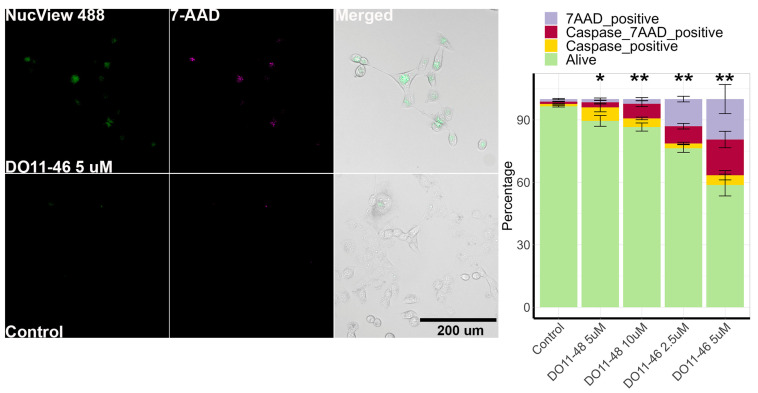
**Cell death induction by DO11-46 and DO11-48.** Images of cells stained with caspase 3/7 probe NucView 488 and dead cell stain 7-AAD. Quantitative analysis provided a graph for H1299 cells treated with 2.5 and 5 µM **DO11-46** or 5 and 10 µM **DO11-48** for 72 h. Experiments were repeated three times, and, for each repeat, 4 random images were taken using automated microscopy, and at least 100 cells were analyzed. * *p*-value < 0.05 and ** *p*-value < 0.01 as determined by two-tailed *t*-test.

To investigate the mechanism of the cytotoxic action of piperazine-substituted pyranopyridines, we evaluated two compounds, namely, **DO11-46** and **DO11-48**, that acted in submicromolar concentrations. The first step was to which type of cell death they induce. This was carried out by staining H1299 cells with 7AAD dye that stains non-viable cells (with damaged membrane) and NucView-488 dye for caspase 3/7 activity as they are activated during apoptosis. Imaging and analysis protocols for H1299 were established earlier [41], and cells were imaged using automated microscopy and then analyzed using Cellpose [42] and CellProfiler [43]. The results show that **DO11-48** and especially **DO11-46** increased the number of apoptotic and necrotic cells (Figure 1). In the case of **DO11-48**, the number of caspase-positive cells exceeded the number of caspase-negative 7AAD-positive cells, indicating that the main type of cell death is apoptosis. However, these two populations were similar during treatment with **DO11-46**, suggesting that this group of compounds could also trigger necrosis. **DO11-46** at 5 µM completely inhibited H1299 cell proliferation and showed more potency than **DO11-48** (Figure S1A).

Next, we evaluated if the compounds affected cell cycle and ERK1/2 activity. For that, we used H1299 with PIP-FUCCI [44] or ERK-KTR fluorescent reporters [45], which allow one to measure cell cycle phases and ERK1/2 activity in live cells using microscopy [46]. Despite the significant inhibition of H1299 proliferation, we did not detect significant changes in either ERK1/2 activity (Figure S1B) or cell cycle progression (Figure S2).

### 2.4. Intracellular ROS Assay

**Figure 2:**
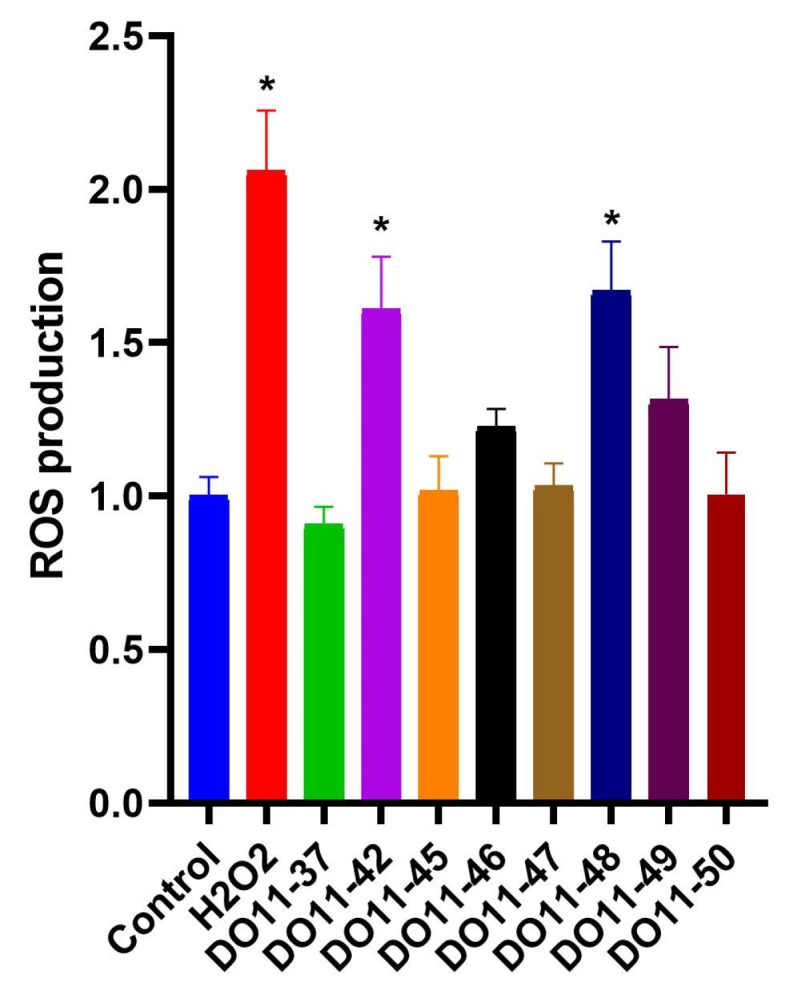
Changes in intracellular ROS level in A549 cells under the treatment of studied compounds. Data are expressed as mean ± SD; * *p* < 0.05 compared to untreated control group.

**Figure 3:**
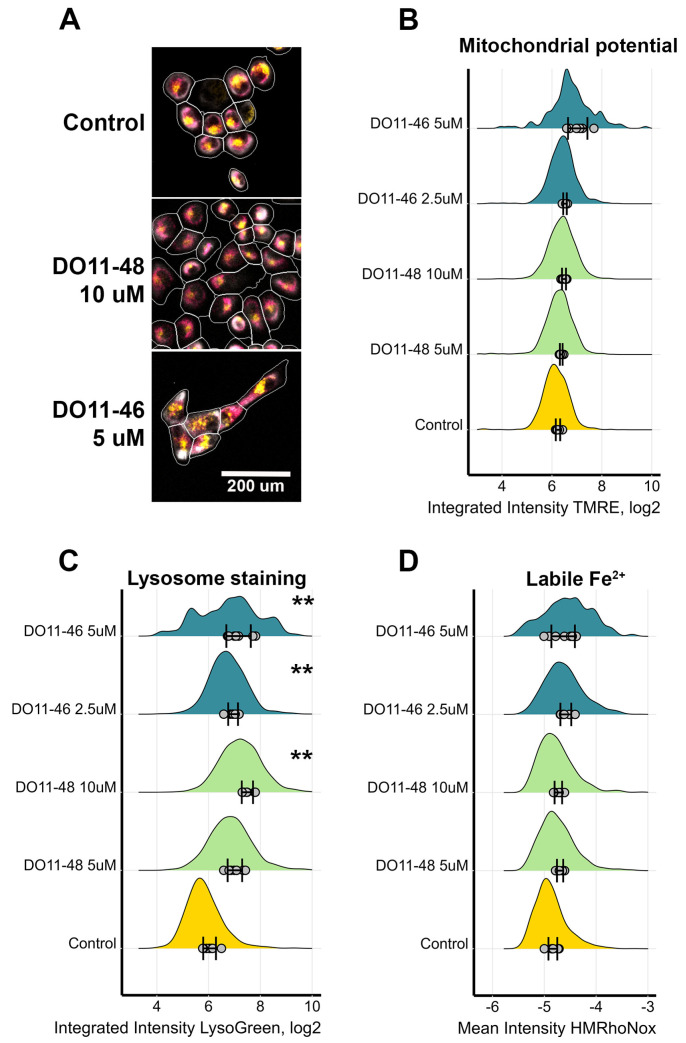
Measurement of mitochondria activity, labile Fe levels, and lysosomes accumulation in H1299 cells. (**A**) Images of cells treated with **DO11-46** and **DO11-48** for 72 h and then stained with potential-dependent mitochondria stain TMRE (magenta), lysosome stain LysoGreen (yellow), and tubulin stain Tubulin Tracker Deep Red (grey). Cell borders were identified using DNA-stain Hoechst-33342 and Tubulin Tracker Deep Red using Cellpose and CellProfiler. Identified cells are outlined in white. (**B**) Distribution of mitochondrial staining with TMRE. (**C**) Distribution of lysosome staining with LysoGreen. (**D**) Distribution of labile Fe^2+^ stained with HMRhoNox-M. Dots represent the mean values for each image, SD for the mean values across different images are shown, and distribution is computed based on overall number of cells, ranging from 500 to 4000 cells. Experiments were repeated three times, and, for each repeat, 4 random images were taken using automated microscopy, and at least 125 cells were analyzed. ** *p*-value < 0.01 as determined by two-tailed *t*-test.

As cell death is often triggered by increased production of reactive oxygen species (ROS), the next step was to evaluate if our compounds act as pro-oxidants. ROS production was accessed using cell-permeable redox sensitive DCFH2-DA dye. The results, presented on Figure 2, demonstrate that only two of the compounds had a statistically significant effect on ROS production, while other toxic compounds did not alter the redox status of cells. As one of the main sites of ROS production is mitochondria, we also evaluated their membrane potential during treatment with either **DO11-46** or **DO11-48**. However, neither of them caused mitochondrial dysfunction (Figure 3A,B). Finally, these two compounds also did not affect levels of Fe^2+^ ions (Figure 3C), increased levels of which often underlines ROS production via Fenton reaction [47]. Both **DO11-46** and **DO11-48** induced the accumulation of lysosomes, as was detected by staining cells with LysoGreen (Figure 3D). Lysosome activation often accompany late stages of apoptosis [48], which is in good accordance with our data on the induction of caspase3/7 activity.

**Figure 4:**
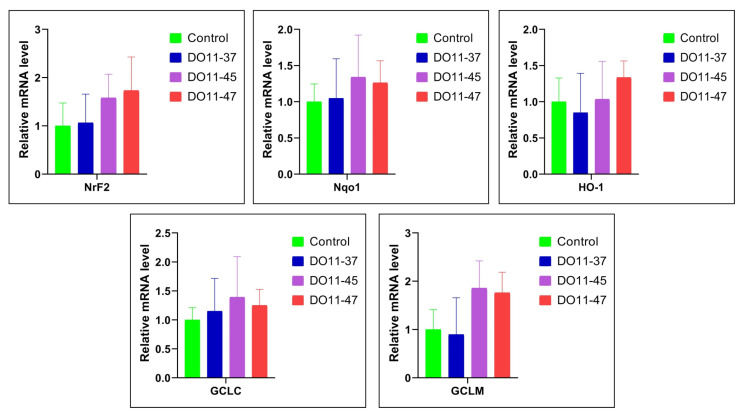
Changes in the expression of Nrf2, Nqo1, HO1, GCLC, and GCLM genes in A549 cells under the treatment of **DO11-37**, **DO11-45**, and **DO11-47**. Data are expressed as mean ± SD.

Increased ROS production is often counteracted by the induction of antioxidant enzymes, many of which are controlled by the Nrf2 transcription factor [49]. So, we analyzed if the compounds could have activated this antioxidant defense system. However, real-time RT-PCR analysis did not reveal the elevation of the mRNA levels of Nrf2 and the downstream genes (Nqo1, HO-1, GCLC, and GCLM) (Figure 4). So, this series of compounds do not act as electrophiles that activate the Nrf2/ARE pathway.

### 2.5. Antiviral Activity

The final step of the project was the evaluation of these eight compounds against influenza A virus, SARS-CoV-2, poliovirus type 1 (PV1), Newcastle disease virus (NDV), herpes simplex virus type 1 (HSV-1), and vaccinia virus (VV), as well as hepatitis B virus (HBV). Compounds were tested at non-toxic and subtoxic concentrations. Unfortunately, most of them did not affect the replication of RNA viruses (Table 3). Weak though statistically significant, a decrease in the replication of influenza virus and SARS-CoV-2 was noted for **DO11-37** and **DO11-46**. However, both of them could reduce virus titers just within one log maximum, which is insufficient for their further evaluation as antiviral agents.

Anti-HBV activity was tested in non-tumor HepaRG^NTPC^ cells pre-infected with the virus to represent a model of chronic infection. The total HBV RNA (tRNA) levels inside the cells (representing levels of transcription) and HBV DNA in conditioned medium (representing virion production) were quantified by real-time PCR. Interestingly, **DO11-37**, **DO11-42**, **DO11-45,** and **DO11-46** significantly reduced levels of virion production even at 1 µM concentration, indicating that their antiviral effect occurs at micromolar concentrations (Table 4). As the same compounds did not alter levels of viral total RNA, they are likely to act at a post-transcriptional step of the virus life cycle.

## 3. Discussion

Chemotherapy, a systemic treatment using chemicals to target rapidly dividing cancer cells, is often limited by non-selectivity, leading to significant toxicity in healthy tissues. Additional challenges include the rapid emergence of drug resistance, molecular instability, and poor solubility, hindering effective cell membrane permeation [50]. These limitations necessitate the development of alternative treatments with improved therapeutic efficacy and reduced side effects [51]. To date, numerous heterocyclic compounds, such as purine, indole, pyrimidine, pyridine, furan, thiazole, pyrrole, quinoline, isoquinoline, pyrrolidine, piperazine, thiolane, thiophene, pyrazine, piperidine, azepine, and chroman, have demonstrated antiproliferative activity against various cancer cell lines [24,52,53]. Specifically, piperazine ranks as the third most significant nitrogen-containing molecule in drug development, with well-established therapeutic properties [24,54,55].

Recent studies have highlighted the potential of piperazine hybrids, such as flavonamidrazones and chalcone-piperazine derivatives, for activity against diverse cancer cell lines including K562, HeLa, and A549 [56]. For instance, a novel series of N^1^-(flavon-7-yl) amidrazones with N-piperazine structures demonstrated potent anticancer activity against leukemic cell lines (including K562). Among the tested compounds, 7-[2-(2-oxo-1-(piperidin-1-yl)propylidene)hydrazinyl]-2-phenyl-4H-chromen-4-one (**11a** in the cited paper) achieved an IC_50_ value of 2.56 ± 0.57 μg/mL [57]. Here, the compound **DO11-48** showed the most pronounced anticancer activity against K562, with an IC_50_ value of 0.5 ± 0.1 μM (Table 2).

Additionally, several chrysin-based sulfonylpiperazines have shown a remarkable inhibition of HeLa cell lines, with IC_50_ values as low as 8.2 μM (4.67 μg/mL) [58]. In our studies, compounds **DO11-42**, **DO11-46**, and **DO11-49** exhibited even stronger activity against HeLa cells, with IC50 values of 1.9 µM, 1.9 µM, and 1.7 µM, respectively (Table 2).

Similarly, Z. Mao et al. demonstrated that chalcone-piperazine hybrids possess antiproliferative activity against various cancer cell lines. Among the tested compounds, **7c** (C_29_H_31_C_l_N_3_O_2_) was the most potent against A549, with an IC_50_ of 5.24 μM [59]. In contrast, another study reported that chalcone-piperazine dimethylamino-4’-[N-(2-oxopropyl)-1-piperazinyl]chalcone exhibited even greater inhibitory activity against the A549 cell line, with an IC_50_ of 0.19 µM [60]. In our experiments with A549 cell lines, **DO11-49** showed the highest activity, achieving an IC_50_ of 2.0 μM (Table 2).

H. Chen et al. investigated a series of 29 arylpiperazine derivatives against human prostate cancer cell lines (PC-3, LNCaP, and DU145). Compounds **7** and **29** were found to exhibit anticancer activity against DU145, with IC_50_ values of 5.8 μM and 7.7 μM, respectively [61]. Meanwhile, novel piperazine derivatives of vindoline demonstrated more significant activity, with [4-(trifluoromethyl)benzyl]piperazine and 1-bis(4-fluorophenyl)methylpiperazine achieving GI_50_ values of 1.6 µM and 1.8 µM, respectively [62]. Among our tested compounds, **DO11-46** demonstrated the most prominent activity against DU145, with an IC_50_ of 0.5 μM. Other compounds, including **DO11-42**, **DO11-48**, and **DO11-49**, also exhibited notable activity against DU145, with IC_50_ values of 1.3 µM, 2.5 µM, and 1.1 µM, respectively (Table 2).

Overall, the anticancer studies revealed that compounds **DO11-42**, **DO11-46**, **DO11-48**, and **DO11-49** have selective inhibitory effects on various cancer cell cultures. Notably, these compounds exhibited lower cytotoxicity against non-tumor HepaRG and SH-SY5Y and the glioblastoma cell lines (Table 2). The structure–activity relationship (SAR) analysis revealed that the presence of electron-withdrawing substituents in the ortho position of the benzene ring enhanced selective anticancer activity, as observed with **DO11-42**, **DO11-46**, **DO11-48**, and **DO11-49**. Conversely, meta-position substituents, as in **DO11-47**, reduced anticancer efficacy. Double substituents in both ortho and meta or para positions further amplified activity, particularly in **DO11-46** and **DO11-49**.

The anticancer effects of these compounds likely involve apoptosis induction, a programmed cell death pathway characterized by caspase activation and mitochondrial cytochrome c release [63]. Additionally, it has been increasingly recognized that conventional chemotherapeutic agents not only trigger apoptosis but also other forms of non-apoptotic cell death, such as necrosis, necroptosis, autophagy, mitotic catastrophe, and senescence [64,65]. The results revealed that both compounds inhibited cell growth through a combination of necrosis and apoptosis. Notably, **DO11-48** predominantly promoted apoptotic cell death, while **DO11-46** exhibited a more balanced effect, with the proportions of caspase-7AAD-positive cells (apoptotic/necrotic) and caspase-negative 7AAD-positive cells (necrotic) being nearly equal. In contrast, the number of caspase-positive cells (purely apoptotic) was significantly lower (Figure 1).

We next investigated the potential mechanisms underlying apoptosis induction by these compounds, including their impact on the cell cycle. Extracellular signal-regulated protein kinases 1 and 2 (ERK1/2), members of the mitogen-activated protein kinase superfamily, are known to mediate cell proliferation and apoptosis [66]. In tumors, ERK1/2 activity has been shown to promote the expression of anti-apoptotic proteins while suppressing pro-apoptotic proteins, thereby supporting cell survival and tumorigenesis [67]. However, our investigation of ERK1/2 activity revealed no significant changes in either ERK1/2 signaling (Figure S1B) or cell cycle progression (Figure S2).

Reactive oxygen species (ROS) are byproducts of normal cellular metabolism and play dual roles: at low to moderate concentrations, ROS contribute to physiological processes, while at elevated levels, they can damage cellular components and induce cell death [68]. Tumors frequently generate high levels of ROS, contributing to DNA damage, genomic instability, and therapy resistance but also potentially promoting apoptosis under specific conditions [69]. Thus, we evaluated the ability of the compounds to generate ROS as a potential mechanism of apoptosis induction. Our studies showed that only **DO11-42** and **DO11-48** significantly increased ROS generation, while other toxic compounds did not affect cell redox status. We also assessed if the compounds trigger mitochondrial disfunction as these organelles are one of the main sources of ROS and play a critical role in intracellular oxidative stress [70]. Mitochondrial ROS can enhance membrane permeability, promote Ca^2+^ uptake, and release cytochrome c, initiating apoptosis [71]. To evaluate mitochondrial involvement, we assessed the mitochondrial membrane potential during treatment with **DO11-46** or **DO11-48**. However, the results did not indicate significant changes or damage to the mitochondrial membrane. So, the antiproliferative activity of the compounds is not mediated via ROS production.

Various agents, including drugs, can induce lysosomal membrane permeability (LMP), leading to the translocation of acidic hydrolases into the cytoplasm and promoting lysosomal-mediated cell death. This form of cell death is particularly significant in anticancer therapy as it can target both cancer cells with defective apoptotic pathways and drug-resistant cells [72]. Our studies demonstrated that **DO11-46** and **DO11-48** induced lysosomal accumulation in H1299 cells. Lysosomes influence apoptosis and autophagic cell death, but extensive research also highlights their role as prerequisites for the execution of regulated necrosis (RN), including necroptosis, ferroptosis, and pyroptosis [73].

LMP, accompanied by the release of hydrolases, is a critical step in initiating RN in most cases. Current evidence suggests that LMP acts as either an initiator or a proteolytic amplifier, triggering the final execution phase of RN [74]. Furthermore, activated caspase-3 and caspase-8 are closely associated with lysosomal membrane permeability [73]. Since our studies indicated that **DO11-46** and **DO11-48** activate caspase-3/7 and induce 7-ADD-positive membrane damage, the accumulation of lysosomes and the subsequent induction of LMP may represent a potential mechanism driving both apoptosis and regulated necrosis in H1299 cells.

In cancer biology, the dysregulation of signaling pathways is a common driver of tumor development, metastasis, invasion, and other malignant processes [75]. The phosphoinositide 3-kinase (PI3K)-protein kinase B (PKB/AKT)-mammalian target of rapamycin (mTOR) axis is a key signaling pathway connecting oncogenes and multiple receptor types, influencing essential cellular functions [76]. The mTOR protein kinase serves as a critical growth-control hub, integrating signals from Ras, PI3K, and growth factors, as well as nutrient inputs. Notably, mutations in components of the Ras and PI3K pathways are prevalent in many human cancers. These mutations contribute to the loss of growth-control checkpoints and enhanced cell survival [77].

The PI3K/AKT/mTOR pathway is frequently activated across a wide range of cancers, including breast, gastric, ovarian, colorectal, prostate, glioblastoma, and endometrial cancers [78]. Consequently, this pathway represents a promising target for pharmacological intervention [79,80]. Inhibition of PI3K has been shown to induce rapid apoptosis, characterized by the cleavage of caspase-3, caspase-7, and PARP [52]. While further studies are necessary, the observed apoptotic activity of **DO11-46** and **DO11-48**, along with their caspase activation profiles, suggests that inhibition of the PI3K/AKT/mTOR pathway may contribute to their mechanism of action.

Compounds with antioxidant and anti-lipoxygenase activity may offer both therapeutic and prophylactic benefits in cancer treatment, particularly for multidrug-resistant tumors, where there is a need for molecules that can target multiple cancer-related pathways [29]. ROS can be scavenged by endogenous antioxidant enzymes, which play a critical role in reducing the risk of oxidative damage [81]. A major protective mechanism against oxidative stress is the activation of antioxidant response elements within cells, controlled primarily by the transcription factor Nrf2. This pathway regulates the expression of genes involved in antioxidant defense and detoxification, such as HO-1, NQO1, GCLC, GCLM, GST, NAD(P)H, GSR, GPX1, TALDO, and MT1E [82,83]. Thus, the Nrf2 pathway is recognized as playing a crucial role in protecting against oxidative stress [84,85]. Compounds **DO11-37**, **DO11-45**, and **DO11-47** demonstrated low cytotoxic effects on various cell lines and low ROS generation. Considering this, we studied their antioxidant effects by investigating the expression of Nrf2, NQO1, HO-1, GCLC, and GCLM genes in A549 cell lines under treatment with these compounds. Treatment with **DO11-45** and **DO11-47** resulted in an increased expression of these genes compared to untreated cells. Although the increase was not statistically significant, it could still contribute to cytoprotection.

Recent studies have highlighted the potential of piperazine analogs, specifically piperazine-modified nucleozins and piperazine-based berberine analogs, as anti-influenza drugs [86,87]. Additionally, N-arylpiperazine derivatives have been investigated as potential IFN inducers [88]. Given these findings, we explored the antiviral properties of eight compounds against influenza A/(H1N1) pdm09 and SARS-CoV-2, PV1, NDV, HSV-1, and VV. However, these compounds did not demonstrate significant efficacy in reducing viral titers in infected cell lines for either of these viruses (Table 3).

One of the most important findings of our study is the antiviral activity of the compounds against hepatitis B virus. HBV is an oncogenic virus that accounts for app 205 mln chronic carriers worldwide [89]. Despite the existence of prophylactic vaccines, this infection still imposes significant threat for healthcare systems as it is responsible for 1.1 mln cases of patients death from end-stage liver disease annually [89]. Anti-HBV therapy is mainly based on nucleos(t)ide analogs that target virus replication [90,91], with several types of new classes of drugs in clinical trials. We show that the compounds **DO11-37**, **DO11-42**, **DO11-45**, and **DO11-46** suppress HBV reproduction at late stages of virus life cycle in submicromolar concentrations, presumably by interfering with capsid assembly. Specifically, **DO11-37** can be considered as lead compound as it has high antiviral activity and very low toxicity, especially towards non-tumor HepaRG cells that resemble human hepatocytes.

## 4. Materials and Methods

### 4.1. Reagents and Cell Lines

#### 4.1.1. Chemistry

General Information: All chemicals and solvents were of commercially high purity grade, used without further purification, and purchased from Sigma-Aldrich (Saint Louis, MO, USA). ^1^H and ^13^C NMR spectra were recorded in DMSO-d_6_/CCl_4_ (1/3) solution (300 MHz for ^1^H and 75.462 MHz for ^13^C, respectively) on a Mercury 300VX spectrometer (Varian Inc., Palo Alto, CA, USA). Chemical shifts are expressed in ppm relative to tetramethylsilane (TMS). Chemical shifts are reported as position (δ in ppm), multiplicity (s = singlet, d = doublet, t = triplet, q = quartet, p = pentet, dd = double doublet, br = broad, and m = multiplet), coupling constant (J in Hz), relative integral, and assignment. The IR spectra were recorded on a Nicolet Avatar 330-FT-IR spectrophotometer (Thermo Nicolet, Vacaville, CA, USA) in vaseline, ν_max_ in cm^−1^. High-resolution mass spectra were recorded on a micrOTOF-Q II device (Bruker Daltonics, Fremont, CA, USA) by electrospray ionization mass spectrometry (ESI-HRMS). Measurements were carried out in positive ion mode; samples were injected into the mass-spectrometer chamber from an HPLC system Agilent 1260 (Agilent Technologies, Santa-Clara, CA, USA). The following parameters were used: capillary voltage 4500 V; mass scanning range: *m*/*z* 50–3000; external calibration with Electrospray Calibrant Solution (Fluka, Darmstadt, Germany); gas pressure 0.4 bar; nitrogen spray gas (4 L/min); interface temperature: 180 °C; flow rate 3 μL/min. Molecular and fragmentation ions in the spectra were analyzed and matched with the appropriately calculated *m*/*z* and isotopic profiles in the Bruker DataAnalysis 4.0 program. Earlier-prepared dry samples were dissolved in 50% acetonitrile in water and injected into the mass-spectrometer spray chamber from an Agilent 1260 HPLC chromatograph equipped with an Agilent Poroshell 120 EC-C18 column (3.0 × 50 mm; 2.7 μm; USA) and a compatible pre-column cartridge using an autosampler. The column was eluted with a mixture of acetonitrile (A) and water (B) in a gradient concentration with a flow rate of 400 μL/min in the following gradient parameters: 0–15% A for 6 min, 15–85% A for 1.5 min, 85–0% A for 0.1 min, and 0% A for 2.4 min. Elemental analysis was performed using a EuroEA 3000 analyzer (Agilent Technologies) by burning a portion of the sample in an oxygen flow followed by chromatography of the combustion gases. Melting points were determined on an MP420 melting point apparatus (Torontech, Toronto, Canada).

#### 4.1.2. Biology

The synthesized compounds were kept as 40 mM stock solutions in DMSO (Sigma-Aldrich).

A549 (CCL-185), HeLa cells (CCL-2), DU145 (HTB-81), K562 (CCL-243), and SH-SY5Y (CRL-2266) cell lines were purchased from American Type Culture Collection (ATCC, Manassas, VA, USA). HEK293T-ΔIFNAR1 cells were described earlier [92]. The low passage cultures of glioblastoma multiforme cells GBM5522 and GBM6138, obtained from surgically removed tumor tissues, were described previously [93,94]. The HepaRG cells were a kind gift of Birke Bartosch (Lyon Cancer Research Center, Lyon, France). H1299 cells were gifted by the Heinrich-Pette Leibniz Institute of Virology (Hamburg, Germany).

DMEM, RPMI, DMEM/F12, and Williams E media were supplied by Gibco (Thermo Scientific, Waltham, MA, USA), and glutamine and trypsin-EDTA were purchased from PanEco (Moscow, Russia). Fetal bovine serum (FBS) was supplied by HyClone (Logan, UT, USA), while FetalClone II by Invitrogen (Carlsbad, CA, USA). The oligonucleotides were synthesized by Evrogen (Moscow, Russia). Evrogen also provided Mint reverse transcriptase and qPCRmix-HS SYBR mixture for real-time PCR.

### 4.2. Synthesis of Compounds

**General Method for the Preparation of Compounds D011-37, D011-42, D011-45, D011-46, D011-47, D011-48, D011-49, and D011-50:** To an aqueous-alcoholic solution of sodium carbonate prepared by dissolving 1.06 g (10 mmol) Na_2_CO_3_ in 20 mL of water and 20 mL of ethanol, add 3.04 *g* (10 mmol) of 3,3-dimethyl-8-piperazine-6-thioxo-3,4,6,7-tetrahydro-1*H*-pyrano[3,4-*c*]pyridine-5-carbonitrile ester (**1**) and stir at room temperature until dissolved. Then, 10 mmol of chloride is added dropwise to the reaction mixture and stirring is continued for another 6 h. The next day, the precipitated crystals are filtered off, washed with water, dried, and recrystallized from ethanol.

NMR and LC-MS spectra of compounds are provided as Supplementary Materials. 2-[(5-Cyano-3,3-dimethyl-8-piperazin-1-yl-3,4-dihydro-1H-pyrano[3,4-c]pyridin-6-yl)thio]acetamide (D011-37): White crystalline powder; yield 83.6%, m.p. 207–208 °C. IR ν/cm^−1^: 1679 (C=O), 2210 (C≡N), and 3113–3443 (NH_2_, NH). ^1^H-NMR (300 MHz, DMSO-d_6_/CCl_4_, 1/3) δ_H_ (ppm): 1.30 (6H, s, 2*CH_3_), 2.68 (2H, s, 4-CH_2_), 2.80 (1H, br, NH), 2.82–2.89 (4H, m, HN(CH_2_)_2_), 3.23–3.29 (4H, m, N(CH_2_)_2_), 3.78 (2H, s, SCH_2_), 4.45 (2H, s, OCH_2_), 6.90 (1H, br, NH_2_) and 7.13 (1H, br, NH_2_). ^13^C NMR (75 MHz, DMSO-d_6_/CCl_4_, 1/3) δ_C_: 26.4 (2*CH_3_), 32.7 (SCH_2_), 37.9 (CH_2_), 45.5 (2*NCH_2_), 49.7 (2*NCH_2_), 58.9 (OCH_2_), 69.3, 97.1 (C-CN), 114.7, 115.0, 147.3, 157.4, 158.1, 168.6 (C=O). Anal. calc. for C_17_H_23_N_5_O_2_S: C, 56.49; H, 6.41; N, 19.38; S, 8.87%. Found: C, 56.77; H, 6.14; N, 19.12; S, 9.08%. HRMS (ESI) of C_17_H_23_N_5_O_2_S, *m*/*z*: calcd for 362.1645 [M + H]^+^, found: 362.1653.

**6-[(2-Chlorobenzyl)thio]-3,3-dimethyl-8-piperazin-1-yl-3,4-dihydro-1H-pyrano[3,4-c]pyridine-5-carbonitrile** (**D011-42**): White crystalline powder; yield 97.2%; mp 171–172 °C. IR *ν*/cm^−1^: 2208 (C≡N), and 3204, 3227 (NH). ^1^H NMR (300 MHz, DMSO-*d_6_*/CCl_4_, 1/3) δ_H_ (ppm): 1.30 (6H, s, 2*CH_3_), 2.68 (2H, s, 4-CH_2_), 2.75 (1H, br, NH), 2.80–2.88 (4H, m, HN(CH_2_)_2_), 3.18–3.25 (4H, m, N(CH_2_)_2_), 4.46 (2H, s, OCH_2_), 4.56 (2H, s, SCH_2_), 7.22–7.27 (2H, m, C_6_H_4_), 7.36–7.42 (1H, m, C_6_H_4_) and 7.48–7.53 (1H, m, C_6_H_4_). ^13^C NMR (75 MHz, DMSO-*d_6_*/CCl_4_, 1/3) δ_C_: 26.3 (2*CH_3_), 31.0 (SCH_2_), 37.9 (CH_2_), 45.3 (2*NCH_2_), 49.6 (2*NCH_2_), 58.8 (OCH_2_), 69.3, 97.4 (C-CN), 114.4, 115.4, 126.6 (CH=), 128.3 (CH=), 128.9 (CH=), 130.1 (CH=), 133.3, 134.4, 147.5, 157.4, 158.1. Anal. Calcd for C_22_H_25_N_4_OSCl: C 61.60; H 5.87; N 13.06; S 7.47; Cl 8.26%. Found: C 61.28 H 5.95; N 13.29; S 7.32; Cl 8.45%. HRMS (ESI) of C_22_H_25_ClN_4_OS, *m*/*z*: calcd for 429.1510 [M + H]^+^, found: 429.1505.

**2-[(5-Cyano-3,3-dimethyl-8-piperazin-1-yl-3,4-dihydro-1H-pyrano[3,4-c]pyridin-6-yl)thio]-N-(3-methylphenyl)acetamide** (**D011-45**): A white solid; yield 97.9%; mp 210–211°C. IR *ν*/cm^−1^: 1652 (C=O), 2210 (C≡N), and 3049, 3119, 3185, 3240 (NH). ^1^H NMR (300 MHz, DMSO-d_6_/CCl_4_, 1/3) δ_H_ (ppm): 1.29 (6H, s, 2*CH_3_), 2.28 (1H, br, NH(CH_2_)_2_), 2.29 (3H, s, PhCH_3_), 2.68 (4H, m, 4-CH_2_), 2.72–2.80 (4H, m, N(CH_2_)_2_), 3.15–3.23 (4H, m, HN(CH_2_)_2_), 4.00 (2H, s, SCH_2_), 4.44 (2H, s, OCH_2_), 7.00–7.06 (2H, m, C_6_H_4_), 7.40–7.46 (2H, m, C_6_H_4_), 9.81 (1H, s, NHC_6_H_4_). ^13^C NMR (300 MHz, DMSO-d_6_/CCl_4_, 1/3) δ_C_: 20.3 (CH_3_), 26.4 (2*CH_3_), 34.0 (SCH_2_), 37.9 (CH_2_), 45.4 (2*NCH_2_), 49.7 (2*NCH_2_), 58.8 (OCH_2_), 69.3, 97.0 (C-CN), 114.7, 115.1, 118.8 (2*CH=), 128.4 (2*CH=), 131.5, 136.3, 147.3, 157.7, 158.2, 164.8 (C=O). Anal. Calcd for C_24_H_29_N_5_O_2_S: C 63.83; H 6.47; N 15.51; S 7.10%. Found: C 63.56; H 6.72; N 15.70; S 6.92%. HRMS (ESI) of C_24_H_29_N_5_O_2_S, *m*/*z*: calcd for 452.2115 [M + H]^+^, found: 452.2129.

2-[(5-Cyano-3,3-dimethyl-8-piperazin-1-yl-3,4-dihydro-1H-pyrano[3,4-c]pyridin-6-yl)thio]-N-(2,5-dichlorophenyl)acetamide (D011-46): A white solid; yield 73.75%; mp 151–152 °C. IR *ν*/cm^−1^: 1683 (C=O), 2204 (C≡N), and 3080–3500 (NH, NH). ^1^H NMR (300 MHz, DMSO-d_6_/CCl_4_, 1/3) δ_H_ (ppm): 1.30 (6H, s, 2*CH_3_), 2.68 (2H, s, 4-CH_2_), 2.75–2.83 (4H, m, NH(CH_2_)_2_), 2.84 (1H, br, NH), 3.02 (1H, br, NH(CH_2_)_2_), 3.14–3.22 (4H, m, N(CH_2_)_2_), 4.01 (2H, s, SCH_2_), 4.44 (2H, s, OCH_2_), 7.36 (1H, d, J = 8.8, 3′-C_6_H_3_), 7.49 (1H, dd, J_1_ = 8.8, J_2_ = 2.5, 4′- C_6_H_3_), 7.90 (1H, d, J = 2.5, 6′-C_6_H_3_), 10.22 (1H, s, NH-Ar). ^13^C NMR (300 MHz, DMSO-d_6_/CCl_4_, 1/3) δ_C_: 26.4 (2*CH_3_), 33.7 (SCH_2_), 37.9 (CH_2_), 45.3 (2*NCH_2_), 49.6 (2*NCH_2_), 58.9 (OCH_2_), 69.4, 97.0 (C-CN), 114.8, 115.5, 118.4 (CH=), 120.3 (CH=), 124.9, 129.7 (CH=), 131.2, 138.7, 147.3, 157.3, 158.2, 165.6 (C=O). Anal. Calcd for C_23_H_25_N_5_O_2_SCl_2_: C 54.55; H 4.98; N 13.83; S 6.33; Cl 14.00%. Found: C 54.83; H 5.14; N 13.61; S 6.19; Cl 14.23%. HRMS (ESI) of C_23_H_25_Cl_2_N_5_O_2_S, *m*/*z*: calcd for [M + H]^+^ 506.1179, found: 506.1186.

2-[(5-Cyano-3,3-dimethyl-8-piperazin-1-yl-3,4-dihydro-1H-pyrano[3,4-c]pyridin-6-yl)thio]-N-(3-methoxyphenyl)acetamide (D011-47): A white solid; yield 95.1%; mp 172–173 °C. IR ν/cm^−1^: 1683 (C=O), 2214 (C≡N), and 3080–3500 (NH). ^1^H NMR (300 MHz, DMSO-d_6_/CCl_4_, 1/3) δ_H_ (ppm): 1.29 (6H, s, 2*CH_3_), 2.68 (2H, s, 4-CH_2_), 2.71 (1H, br, NH(CH_2_)_2_), 2.72–2.80 (4H, m, NH(CH_2_)_2_), 3.14–3.22 (4H, m, N(CH_2_)_2_), 3.76 (3H, s, OCH_3_), 4.01 (2H, s, SCH_2_), 4.44 (2H, s, OCH_2_), 6.51–6.56 (1H, m, 4′-C_6_H_4_), 7.02–7.15 (2H, m, 5′,6′-C_6_H_4_), 7.28–7.31 (1H, m, 2′-C_6_H_4_), 9.91 (1H, s, NHAr). ^13^C NMR (300 MHz, DMSO-d_6_/CCl_4_, 1/3) δ_C_: 26.4 (2*CH_3_), 34.1 (SCH_2_), 37.9 (CH_2_), 45.4 (2*NCH_2_), 49.7 (2*NCH_2_), 54.4 (OCH_3_), 58.8 (OCH_2_), 69.3, 97.0 (C-CN), 104.5 (CH=), 108.4 (CH=), 111.0 (CH=), 114.7, 115.2, 128.6 (CH=), 140.0, 147.3, 157.6, 158.2, 159.2, 165.1 (C=O). Anal. Calcd for C_24_H_29_N_5_O_3_S: C 61.65; H 6.25; N 14.98; S 6.86%. Found: C 61.33; H 6.03; N 15.16; S 7.02%. HRMS (ESI) of C_24_H_29_N_5_O_3_S, *m*/*z*: calcd for 468.2064 [M + H]^+^, found: 468.2064.

2-[(5-Cyano-3,3-dimethyl-8-piperazin-1-yl-3,4-dihydro-1H-pyrano[3,4-c]pyridin-6-yl)thio]-N-(2-methoxyphenyl)acetamide (D011-48): A white solid; yield 95.8%; mp 175–176 °C. 1656 (C=O), 2213 (C≡N), and 3213–3519 (NH, NH). ^1^H NMR (300 MHz, DMSO-d_6_/CCl_4_, 1/3) δ_H_ (ppm): 1.30 (6H, s, 2*CH_3_), 2.70 (2H, s, 4-CH_2_), 2.77–2.83 (4H, m, NH(CH_2_)_2_), 2.91 (1H, br, NH(CH_2_)_2_), 3.20–3.27 (4H, m, N(CH_2_)_2_), 3.82 (3H, s, OCH_3_), 4.04 (2H, s, SCH_2_), 4.45 (2H, s, OCH_2_), 6.80–7.01 (3H, m, C_6_H_4_), 8.11–8.16 (2H, m, 6′-C_6_H_4_), 9.02 (1H, s, NHAr). ^13^C NMR (300 MHz, DMSO-d_6_/CCl_4_, 1/3) δ_C_: 26.4 (2*CH_3_), 33.8 (SCH_2_), 38.0 (CH_2_), 45.3 (2*NCH_2_), 49.5 (2*NCH_2_), 55.2 (OCH_3_), 58.9 (OCH_2_), 69.4, 97.4 (C-CN), 110.0 (CH=), 114.5, 115.6, 119.6 (CH=), 120.1 (CH=), 123.21 (CH=), 127.1, 147.6, 147.9, 156.6, 158.2, 165.1 (C=O). Anal. Calcd for C_24_H_29_N_5_O_3_S: C 61.65; H 6.25; N 14.98; S 6.86%. Found: C 61.27; H 6.04; N 15.16; S 7.03%. HRMS (ESI) of C_24_H_29_N_5_O_3_S, *m*/*z*: calcd for 468.2062 [M + H]^+^, found: 468.2059.

2-[(5-Cyano-3,3-dimethyl-8-piperazin-1-yl-3,4-dihydro-1H-pyrano[3,4-c]pyridin-6-yl)thio]-N-(2,5-dimethoxyphenyl)acetamide (D011-49): A white solid; yield 78.8%; mp 173–174 °C. 1656 (C=O), 2214 (C≡N), and 3169–3509 (NH, NH). ^1^H NMR (300 MHz, DMSO-d_6_/CCl_4_, 1/3) δ_H_ (ppm): 1.30 (6H, s, 2*CH_3_), 2.70 (2H, s, 4-CH_2_), 2.76–2.84 (4H, m, NH(CH_2_)_2_), 2.89 (1H, br, NH(CH_2_)_2_), 3.19–3.26 (4H, m, N(CH_2_)_2_), 3.75 (3H, s, OCH_3_), 3.79 (3H, s, OCH_3_), 4.01 (2H, s, SCH_2_), 4.45 (2H, s, OCH_2_), 6.40 (1H, dd, J_1_ = 8.8, J_2_ = 2.6, 5′-C_6_H_3_), 6.47 (1H, d, J = 2.6, 3′-C_6_H_3_), 7.93 (1H, d, J = 8.8, 6′-C_6_H_3_), 8.85 (1H, s, NHAr). ^13^C NMR (300 MHz, DMSO-d_6_/CCl_4_, 1/3) δ_C_: 26.4 (2*CH_3_), 33.7 (SCH_2_), 38.0 (CH_2_), 45.4 (2*NCH_2_), 49.7 (2*NCH_2_), 54.8 (OCH_3_), 55.3 (OCH_3_), 58.9 (OCH_2_), 69.4, 97.3 (C-CN), 98.2 (CH=), 103.5 (CH=), 114.6, 115.5, 120.4, 120.8 (CH=), 147.5, 149.4, 156.0, 156.8, 158.2, 164.7 (C=O). Anal. Calcd for C_25_H_31_N_5_O_4_S: C 60.34; H 6.28; N 14.07; S 6.44%. Found: C 60.72; H 6.03; N 13.95; S 6.61%. HRMS (ESI) of C_25_H_31_N_5_O_4_S, *m*/*z*: calcd for [M + H]^+^ 498.2170, found: 498.2164.

N-(4-acetylphenyl)-2-[(5-cyano-3,3-dimethyl-8-piperazin-1-yl-3,4-dihydro-1H-pyrano[3,4-c]pyridin-6-yl)thio]acetamide (D011-50): A white solid; yield 83.5%; mp 134–135 °C. 1702 (C=O), 2211 (C≡N), and 3275, 3481 (NH, NH). ^1^H NMR (300 MHz, DMSO-d_6_/CCl_4_, 1/3) δ_H_ (ppm): 1.28 (6H, s, 2*CH_3_), 2.52 (3H, s, CH_3_CO), 2.68 (2H, s, 4-CH_2_), 2.70–2.77 (4H, m, NH(CH_2_)_2_), 3.15 (1H, br, NH(CH_2_)_2_), 3.16–3.23 (4H, m, N(CH_2_)_2_), 4.08 (2H, s, SCH_2_), 4.44 (2H, s, OCH_2_), 7.68–7.73 (2H, m, C_6_H_4_), 7.85–7.90 (2H, m, C_6_H_4_), 10.44 (1H, s, NHC_6_H_4_). ^13^C NMR (300 MHz, DMSO-d_6_/CCl_4_, 1/3) δ_C_: 25.9 (CH_3_), 26.4 (2*CH_3_), 34.2 (SCH_2_), 37.9 (CH_2_), 45.0 (2*NCH_2_), 49.1 (2*NCH_2_), 58.8 (OCH_2_), 69.4, 96.9 (C-CN), 114.9, 115.3, 118.0 (2*CH=), 129.0 (2*CH=), 131.5, 143.2, 147.5, 157.5, 158.0, 165.9 (C=O), 195.3 (C=O). Anal. Calcd for C_25_H_29_N_5_O_3_S: C 62.61; H 6.09; N 14.60; S 6.69%. Found: C 62.85; H 5.91; N 14.36; S 6.88% HRMS (ESI) of C_25_H_29_N_5_O_3_S, *m*/*z*: calcd for 480.2064 [M + H]^+^, found: 480.2055.

### 4.3. Cell Cultivation and Antiproliferative Activity

HeLa, DU145, SH-SY5Y, H1299, HEK293T-ΔIFNAR1, GBM5522, and GBM6138 cells were maintained in DMEM, whereas A549 cells in DMEM/F12, supplemented with 10% FBS and 2 mM glutamine. The cells were grown at 37 °C in a humid atmosphere containing 5% CO_2_ and split 2–3 times a week. All cell lines were regularly tested for mycoplasma by PCR. Sorafenib and 5-fluorouracil were obtained from Sigma.

The control non-tumor HepaRG cell line was cultivated in Williams E medium supplemented with 10% Fetal Clone II, 5 µg/mL insulin, 50 µM hydrocortisone, 50 U/mL penicillin, and 50 µg/mL streptomycin (the “Complete” medium). The cells at 95% density were referred to as HepaRG^undiff^. The cells were differentiated as described in [38]. Briefly, they were seeded on 48 or 6-well plates, grown without splitting for 2 weeks after monolayer formation in the “Complete” medium and then for an additional 2 weeks in the same medium supplemented with DMSO. The resulting culture was referred to as HepaRG^diff^.

The cells with the exception of HepaRG line were seeded on a 96-well plate at a 5000 cells/well density. Twenty-four hours later, the compounds were added to 1, 5, 10, 25, 75, and 100 µM concentrations (the final concentration of DMSO in the medium did not exceed 0.0001%). After 72 h incubation, cell viability was assessed by standard 3-(4, 5-dimethylthiazolyl-2)-2, 5-diphenyltetrazolium bromide reagent [95]. In the case of SH-SY5Y cells, viability was evaluated by the crystal violet staining method [96]. In both cases, optical density (OD) was measured at 544 nm on a CHAMELEON V microplate reader (Hidex Ltd., Turku, Finland). Cell proliferation was calculated as a mean OD of treated wells to mean OD of control wells and expressed in per cents. For each compound, an IC_50_ value was calculated as its concentration at which this compound suppressed cell growth rate by 50% (IC_50_). For HepaRG cells, IC50 values represented the concentration at which the respective compounds decreased number of viable cells by 50%.

### 4.4. Quantification of Production of Reactive Oxygen Species

Pro-oxidant activity of the compounds was studied by quantification of ROS production using a cell-permeable fluorogenic probe 2′,7′-dichlorodihydrofluorescein diacetate (DCFH2-DA) [97,98]. A549 cells were seeded on a 48-well plate at a density of 10,000 cells/well. Twenty-four hours later, the compounds were added to 10 µM concentration in quadruplicate, and the cells were kept for 72 h. As a positive control, A549 cells were treated with 1 mM H_2_O_2_ for 2 h prior analysis. Then, media were replaced with the fresh FBS-lacking DMEM supplemented with 25 µM DCFH2-DA. After 45 min incubation, the cells were washed 10 times with 500 µL PBS, resuspended in 200 µL PBS and transferred to a black 96-well plate. The fluorescence intensities (FLIs) were measured using CHAMELEON V microplate reader with excitation at 485 nm and emission at 535 nm.

### 4.5. Status of Nrf2/ARE Pathway

A possible effect of the least-toxic 3 compounds (**DO11-37**, **DO11-45**, and **DO11-47**) on the status of Nrf2/ARE pathway was analyzed by quantification of expression of Nrf2 and Nrf2-dependend genes using real-time PCR analysis. A549 cells were seeded in 6-well plates at a density of 3.09 × 10^5^ cells/well in DMEM-F12 media supplemented with FBS and glutamine and treated with 10 µM compounds for three days. Next, total RNA was isolated using CleanRNA standard kit (Evrogen) with subsequent treatment with DNAseI (Roche, Basel, Switzerland), and 2 µg of RNA from each sample was reverse-transcribed using random hexamer primer and mint reverse transcriptase according to manufacturer’s protocol and subjected to real-time PCR as described previously [99] using primers listed in the Table 5.

### 4.6. Mitochondrial Membrane Potential and Lysosome Aggregation

H1299 cells were seeded at 750 cells/well density in 96-well plate, treated with drugs for 72 h and then stained with 1 ug/mL Hoechst-33342 (Lumiprobe, Moscow, Russia), 100 nM TMRE (Lumiprobe, Russia) potential-dependent mitochondria stain, 100 nM LumiTracker LysoGreen (Lumiprobe, Russia) stain for acidic lysosomes, and 0.33 ug/mL Tubulin Tracker Deep Red (Invitrogen, USA). Cells were stained at 37 °C for 30 min with all stains, except for LysoGreen, which was added 5 min before imaging. Cells were washed once and imaged in DMEM with low autofluorescence (BioinnLabs, Rostov na Donu, Russia) with Leica DMI8 automated microscope (Wetzlar, Germany). Fluorescent imaging was performed using 10× objective with the following parameters: for Hoechst-33342 excitation 325–375 nm and emission 435–485 nm, LysoGreen 460–500/512–542 nm, TMRE 541–551/565–605 nm, and Tubulin Tracker 590–650/662–738 nm. For nuclei segmentation, we applied cyto3 model from Cellpose v3 [42] to Hoechst-33342 images, and cell cytoplasm was segmented using merged TMRE and Tubulin Tracker images with Sauvola propagation method in CellProfiler v4.2 [43]. Integrated intensity of TMRE and LysoGreen fluorescence for each cell were calculated using CellProfiler. Detailed protocol for cell segmentation and Python (v.3.11.5) scripts for image quality control and data processing are described in [41]. Experiments were repeated two independent times, and 4 random images were recorded each time.

### 4.7. Cell Death

To analyze cell death mechanisms, we stained cells NucView 488 (Biotium, Fremont, CA, USA) probe for caspase 3 and 7 activity, 1 ug/mL of non-permeable DNA dye 7-AAD (BioinnLabs, Russia), and stain for labile Fe2+ HMRhoNox-M (Lumiprobe, Russia). Cells were treated with drugs for 72 h and then stained for 30 min with 2 uM NucView 488 and 2 uM HMRhoNox-M at 37 °C. Then, cells were washed once in DMEM with low autofluorescence, then 1 ug/mL 7-AAD was added, and cells were imaged using Leica DMI8 automated microscope. Fluorescent imaging was performed using 10x objective with the following parameters: for Hoechst-33342 excitation 325–375 nm and emission 435–485 nm, NucView 488 460–500/512–542 nm, HMRho-Nox-M 541–551/565–605 nm, and 7-AAD 590–650/662–738 nm. Cells were segmented using images in bright field with cyto3 model from Cellpose and then processed in CellProfiler. 7-AAD and NucView 488 images were used to segment nuclei of necrotic or apoptotic cells in CellProfiler. If a cell contained nucleus stained with NucView 488, it was considered as apoptotic, and if it contained a nucleus stained only with 7-AAD, it was considered as necrotic. HMRhoNox-M signal was analyzed as mean fluorescence intensity per cell. Experiments were repeated two independent times, and 4 random images were recorded each time.

### 4.8. Cell Cycle and ERK1/2 Activity Mesurements

The fluorescent reporter cell line H1299 PIP-FUCCI was obtained by lentiviral transfection. The stocks containing VSV-G pseudotyped lentiviral particles were generated by cotransfection of HEK293T with pLenti-CMV-Blast-PIP-FUCCI (Addgene #138715) and packaging plasmids. For the creation of expressing PIP-FUCCI, H1299 cells were transduced with lentiviral particles to achieve ~30 to 50% transduction rate, and then transduced cells were selected with medium supplemented with 5 μg/mL blasticidin. Cells were imaged on 96-well plates using motorized Leica DMI8 fluorescence microscope (Leica, Germany) using 10× magnification lenses. PIP-mVenus were imaged with excitation 460–500 nm and emission in 512–542 nm, and Gem-mCherry were imaged with excitation 541–551 nm and emission in 565–605 nm. Cells were grouped by cell cycle phases according to gates configured by comparing PIP-FUCCI reporter data and cell staining using EdU(5-ethynyl 2’-deoxyuridine)-Hoechst33342 for cells treated with DMSO for 72 h.

To measure ERK1/2 activity, we used H1299 cells with stable ERK-KTR-mClover (Addgene #59150) and H2B-mRuby (Addgene #90236) expression. ERK-KTR allows one to measure ERK1/2 activity by comparing fluorescent protein intensity in cytoplasm and nucleus. Nuclei were segmented in CellProfiler using H2B-mRuby fluorescence. ERK-KTR-mClover was imaged using excitation 460–500 nm and emission in 512–542 nm and H2B-mRuby using 541–551 nm and emission in 565–605 nm. Then, nuclei were expanded by 20 pixels to measure ERK-KTR reporter intensity in cytoplasm. ERK1/2 activity was calculated as the ratio of median signal in cytoplasm to a nucleus for each cell. The cell line and cell segmentation protocols were established earlier in [46]. The number of nuclei was also used to measure H1299 cell proliferation.

Plate layout and autofocus were carried out using LAS X software, version 3.5.7. For each well (biological repeat of drug treatment), four fields were automatically selected with the same pattern for all wells. Image quality control was performed manually by two researches. Images with low quality or presence of optical obstacles (areas with high background signal and serum debris) were excluded from analysis. Images were segmented using Cellpose v3, and fluorescence intensity data were obtained using CellProfiler v4. Image analysis data were processed using custom Python scripts and visualized using ggplot2 in R (version 4.2.3).

### 4.9. Antiviral Activity

#### 4.9.1. Influenza A Virus and SARS-CoV-2

Production of influenza A virus (H1N1) stock and its infection of MDCK cells, as well as infection of Vero E6 cells with SARS-CoV-2, were described previously [100]. Cytotoxicity of the compounds towards MDCK and VeroE6 cells was assessed by seeding 18,000 cells/well of 24-well plates in 0.1 mL DMEM, treating with the compounds for 66 h, and measuring cell viability by MTS assay [101]. As positive controls, oseltamivir and N-hydroxicytidine were used (obtained from Sigma).

The compounds were added at non-toxic concentrations in serum-free DMEM to the respective cells; one hour later, the serial 10-fold dilutions of influenza virus or SARS-CoV-2 were added in serum-free medium, and antiviral activity was determined by the reduction in the infectious titer of the virus by cytopathic action (CPE). Calculation of 50% cytotoxic concentration (CC_50_) and 50% effective concentration (IC50) was performed by standard methods using Microsoft Excel 5.0 and GraphPad Prism 8 (GraphPad Software Inc., Boston, MA, USA).

#### 4.9.2. Hepatitis B Virus (HBV)

Antiviral activity of compounds towards HBV was studied in HepaRG^NTCP^ cells. These cells were differentiated as described previously [102]. Briefly, they were seeded in 12-well plates at a density of 10^5^ cells/well in Williams E medium supplemented with 10% FetalClone II, 2 mM glutamine, 5 µg/mL insulin, and 50 µM hydrocortisone (Sigma, Darmstadt, Germany) (“Complete medium”). Four days after formation of a monolayer, the medium was replaced with complete medium supplemented with 1.8% DMSO (Sigma). Another three days later, the medium was replaced with complete medium supplemented with 1.8% DMSO and 1 µg/mL tetracycline, and the cells were kept for additional 24 h. Then, the cells were infected with 650 genomic equivalents of HBV per cell in complete medium with DMSO and tetracycline supplemented also with 4% polyethylene glycol 8000. Another 24 h later, the medium was removed, and the cells were washed three times with sterile phosphate-buffer saline (PBS), then grown in complete medium with 1.8% DMSO for 5 days, and later treated with the compounds or control ant-HBV agent Lamivudine (3TC) purchased from Sigma for 6 days. After the treatment, conditioned medium was harvested and subjected to purification of HBV genomic DNA using NK-probe kit (DNA Technology, Moscow, Russia), while total RNA was isolated from harvested cells by RNA Extract reagent (Evrogen). Total HBV RNA levels were assessed in samples from cells, and genomic HBV DNA in samples from conditioned medium using primers listed in Table 1 according to Alfaiate et al. [103].

#### 4.9.3. Herpes Simplex Virus (HSV-1), Poliovirus (PV), and Newcastle Disease Virus (NDV)

HEK293T-ΔIFNAR1 cells were seeded on 96-well plated at a density of 1 × 10^4^ cells/well in DMEM supplemented with 10% FBS and 24 h later infected with HSV-1, poliovirus type 1 (PV1), or NDV at multiplicity of infection from 0.001 to 100 with 10-fold dilution in DMEM containing 0.5% FBS in the presence of tested compounds at various concentrations. Two days post-infection, cytopathogenic effect of the viruses was assessed by staining cells with resazurin and quantification of fluorescence at λex 544 nm and λem 590 nm.

#### 4.9.4. Vaccinia Virus (VV)

HEK293T-ΔIFNAR1 cells were seeded on 96-well plated at a density of 1x10^4^ cells/well in DMEM supplemented with 10% FBS and 24 h later infected with vaccinia virus harboring luciferase gene [104] as described above for HSV-1. Two days later, the cells were either stained with resazurin to determine cytopathogenic effect or subjected to luciferase quantification by Promega Luciferase Assay System (Madison, USA) as a readout of virus replication.

### 4.10. Statistical Analysis

All experiments were performed in triplicate at least three times independently. The values are presented as the means ± standard deviations (S.D.). The statistical significance of differences between the two groups was assessed by Student’s *t*-test using GraphPad Prism 8 (GraphPad Software Inc., Boston, MA, USA). Multiple comparisons were evaluated by analysis of variance (ANOVA) with Dunnett’s post hoc test.

## 5. Conclusions

In this study, we have developed two types of compounds. The first lead compound is the non-toxic inhibitor of hepatitis B virus replication that suppressed virion assembly/release. The second type is represented by compounds that exhibit anticancer activity towards a spectrum of tumor cell lines in submicromolar concentrations and is moderately selective towards non-transformed human cells. These lead compounds can be used for the development of novel anticancer and antiviral drugs.

## Figures and Tables

**Table 1:** Yields and functional groups of (5-cyano-3,3-dimethyl-8-piperazin-1-yl3,4-dihydro-1H-pyrano[3,4-c]pyridin-6-yl)thio derivatives.

Compounds N	R	Yield (%) *
**DO11-37**	CH_2_CONH_2_	83.6
**DO11-42**	CH_2_-2-ClC_6_H_4_	97.2
**DO11-45**	CH_2_CONH-4-MeC_6_H_4_	97.9
**DO11-46**	CH_2_CONH-2,5-Cl_2_C_6_H_3_	73.8
**DO11-47**	CH_2_CONH-3-OMeC_6_H_4_	95.1
**DO11-48**	CH_2_CONH-2-OMeC_6_H_4_	95.8
**DO11-49**	CH_2_CONH-2,4-(OMe)_2_C_6_H_3_	78.8
**DO11-50**	CH_2_CONH-4-COMeC_6_H_4_	83.5

* Yield after recrystallization.

**Table 2:** IC_50_ of compounds in different cell lines.

Compound	IC_50_ (µM)							
	A549	HeLa	DU145	K562	SH-SY5Y	GBM5522	GBM6138	HepaRG
**DO11-37**	294.2 ± 35.9	133.2 ± 38.0	225.3 ± 52.4	91.4 ± 29.6	99.4 ± 21.4	309.4 ± 63.3	571.4 ± 77.7	967.9 ±87.8
**DO11-42**	7.0 ± 1.9	1.9 ± 0.3	1.3 ± 0.2	1.5 ± 0.8	4.5 ± 1.8	4.2 ± 1.3	51.2 ± 10.7	24.5 ±2.8
**DO11-45**	22.4 ± 6.8	21.9 ± 12.7	27.7 ± 0.6	5.6 ± 4.4	43.1 ± 11.0	13.5 ± 4.7	129.1 ± 52.8	73.7 ±15.5
**DO11-46**	6.4 ± 0.8	1.9 ± 0.2	0.5 ± 0.6	1.7 ± 0.7	19.8 ± 2.2	3.2 ± 1.5	25.0 ± 12.0	15.2 ± 3.9
**DO11-47**	67.8 ± 5.8	39.3 ± 16.9	10.0 ± 2.9	12.8 ± 4.2	36.7 ± 18.2	20.0 ± 6.8	103.8 ± 22.1	437.2 ± 14.8
**DO11-48**	3.5 ± 0.7	2.2 ± 0.6	2.5 ± 1.2	0.5 ± 0.1	18.1 ± 5.1	3.8 ± 1.5	27.9 ± 2.6	10.9 ± 1.6
**DO11-49**	2.0 ± 0.5	1.7 ± 0.4	1.1 ± 0.3	13.8 ± 3.8	13.3 ± 3.7	4.1 ± 0.4	3.8 ± 1.2	10.6 ± 1.2
**DO11-50**	45.8 ± 12.6	74.5 ± 3.5	26.7 ± 8.4	45.6 ± 20.0	13.2 ± 4.3	15.9 ± 4.2	54.7 ± 28.7	42.4 ± 8.3
**5-Fluorouracil**		1.2 ±0.3		1.3 ± 0.3	1.5 ± 0.2			
**Sorafenib**					40 ± 10	84 ± 14		>100

**Table 3:** Antiviral activity of compounds against RNA and DNA viruses.

Compound	Influenza A Virus			SARS-CoV-2		PV1	NDV	HSV-1	VV
	Δlg_max_	IC_50_, µM	CC_50_, µM	Δlg_max_	IC_50_, µM	IC_50_, µM	IC_50_, µM	IC_50_, µM	IC_50_, µM
**DO11-37**	0.5	>0.3	1.68	0.5	>200	>300	>300	>300	>300
**DO11-42**	0.5	>0.019	0.44	0	>15	>7.5	>7.5	>7.5	>7.5
**DO11-45**	0	>0.038	0.82	0	>30	>30	>30	>30	>30
**DO11-46**	0.5	>0.019	0.40	1.0	>8	>6	>6	>6	>6
**DO11-47**	0	>0.038	0.81	0	> 38	>60	>60	>60	>60
**DO11-48**	0	>0.019	0.83	0	>10	>3.75	>3.75	>3.75	>3.75
**DO11-49**	0	>0.3	0.90	0	>10	>3	>3	>3	>3
**DO11-50**	0	>0.3	1.27	0	>15	>5	>5	>5	>5
**N-HydroxyC**				5.0	0.5				
**Oseltamivir**	6.0	0.02	>10						

**Table 4:** Antiviral activity of compounds against hepatitis B virus (HBV).

Compound	Extracellular HBV DNA (Δlog_10_)		Intracellular Total HBV RNA (Δlog_10_)	
	1 µM	10 µM	1 µM	10 µM
**DO11-37**	1.97 ± 0.03	2.32 ± 0.16	<0.5	<0.5
**DO11-42**	2.52 ± 0.36	2.31 ± 0.29	<0.5	0.53 ± 0.31
**DO11-45**	2.92 ± 0.44	3.31 ± 0.25	<0.5	<0.5
**DO11-46**	2.61 ± 0.43	3.27 ± 0.14	<0.5	<0.5
**DO11-47**	<0.5	<0.5	<0.5	<0.5
**DO11-48**	<0.5	<0.5	<0.5	<0.5
**DO11-49**	<0.5	<0.5	<0.5	<0.5
**DO11-50**	<0.5	<0.5	<0.5	<0.5
**3TC**	0.93	1.04	<0.5	<0.5

**Table 5:** Oligonucleotides used in real-time PCR.

Gene ID	Orientation	Sequence
Nrf2	Forward	5′-TACTCCCAGGTTGCCCACA-3′
	Reverse	5′-CATCTACAAACGGGAATGTCTGC-3′
Nqo1	Forward	5′-CCGTGGATCCCTTGCAGAGA-3′
	Reverse	5′-AGGACCCTTCCGGAGTAAGA-3′
HO-1	Forward	5′-CCAGCAACAAAGTGCAAGATTC-3′
	Reverse	5′-TCACATGGCATAAAGCCCTACAG-3′
GCLC	Forward	5′-GGATTTGGAAATGGGCAATTG-3′
	Reverse	5′-CTCAGATATACTGCAGGCTTGGAA-3′
GCLM	Forward	5′-TGCAGTTGACATGGCCTGTT-3′
	Reverse	5′-TCACAGAATCCAGCTGTGCAA-3′
HBV tRNA	Forward	5′-GCTGACGCAACCCCCACT-3′
	Reverse	5′-AGGAGTTCCGCAGTATGG-3′
HBV DNA	Forward	5′-AAATTCGCAGTCCCCAACCT-3′
	Reverse	5′-CGCAGACACATCCAGCGATA-3′
GUS	Forward	5′-CGTGGTTGGAGAGCTCATTTGGAA-3′
	Reverse	5′-ATTCCCCAGCACTCTCGTCGGT-3′

## Data Availability

The raw data supporting the conclusions of this article will be made available by the authors on request.

## Supplementary Materials

The following supporting information can be downloaded at https://www.mdpi.com/article/10.3390/ijms26093991/s1.

## Abbreviations

The following abbreviations are used in this manuscript:

COVID-19	Coronavirus disease 19
HIV	Human immunodeficiency virus
NCI	National cancer institute
IC_50_	Inhibitory concentration
ERK	Extracellular signal-regulated protein kinase
SD	Standard deviation
ROS	Reactive oxygen species
Nrf2	Nuclear factor erythroid 2-related factor 2
Nqo1	NAD(P)H:quinone dehydrogenase 1
HO-1	Heme oxygenase 1
GCLC	Glutamate–cysteine ligase catalytic subunit
GCLM	Glutamate–cysteine ligase modifier subunit
ARE	Antioxidant response element
PV-1	Poliovirus type 1
NDV	Newcastle disease virus
HSV-1	Herpes simplex virus type 1
VV	Vaccinia virus
SAR	Structure–activity relationship
LMP	Lysosomal membrane permeability
RN	Regulated necrosis
PI3K	Phosphoinositide 3-kinase
mTOR	Mammalian target of rapamycin
TMS	Tetramethylsilane
ESI-MS	Electrospray ionization mass spectrometry
