# Supplementary material for: Piperazine-Substituted Pyranopyridines Exhibit Antiproliferative Activity and Act as Inhibitors of HBV Virion Production
Source: Int J Mol Sci. 2025 Apr 23;26(9):3991. doi: 10.3390/ijms26093991 (PMC12071598; doi:10.3390/ijms26093991)
Supplement: Supplementary file 1 [file ijms-26-03991-s001.zip › ijms-3496671-supplementary.pdf]

SUPPLEMENT

Piperazine-substituted pyranopyridines exhibit antiproliferative activity and act as inhibitors of HBV virion production

Sona Buloyan [1,#] , Arpine Harutyunyan [1,*,#] , Hrachik Gasparyan [1], Anahit Sakeyan [1], Astghik Shahkhatuni [1], Natalia F. Zakirova [2], Gaukhar Yusubalieva [3,2], Ilya M. Kirillov [4], Irina T. Fedyakina [4], Pavel N. Solyev [2], Anastasia V. Lipatova [2], Mikhail A. Bogomolov[2], Vladimir S. Prassolov [2], Timofey D. Lebedev [2], and Alexander V. Ivanov [2,*]

Scientific Technological Center of Organic and Pharmaceutical Chemistry National Academy of Sciences of the Republic of Armenia, Yerevan, Armenia; sonabuloyan@gmail.com (S.B.), hrachikgasparyan@mail.ru (H.G.), harutyunyanarpine@stcopc.sci.am, harutyunyan_arpi@mail.ru (A.H.), anahit.saqeyan.98@mail.ru (A.S.), astriksh@gmail.com (A.Sh.)

[2] Engelhardt institute of molecular biology, Russian Academy of sciences, 119991 Moscow, Russia; nat_zakirova@mail.ru (N.Z.), solyev@gmail.com (P.S.); lipatovaanv@gmail.com (A.L.), bogomolov_mikhail@bk.ru (M.B.), prassolov45@mail.ru (V.P.), lebedevtd@gmail.com (T.L.), aivanov@yandex.ru (A.I.)

[3] Federal Research and Clinical Center of Specialized Medical Care and Medical Technologies FMBA of Russia, 115682 Moscow, Russia, gaukhar@gaukhar.org

[4] Gamaleya National Research Centre for Epidemiology and Microbiology of the Ministry of Russia, 123098 Moscow, Russia; iliyakirillov@yandex.ru (I.K.), irfed2@mail.ru (I.F.)

* Correspondence: aivanov@yandex.ru (A.I.) or harutyunyan_arpi@mail.ru (A.H.)

[#] These authors contributed equally

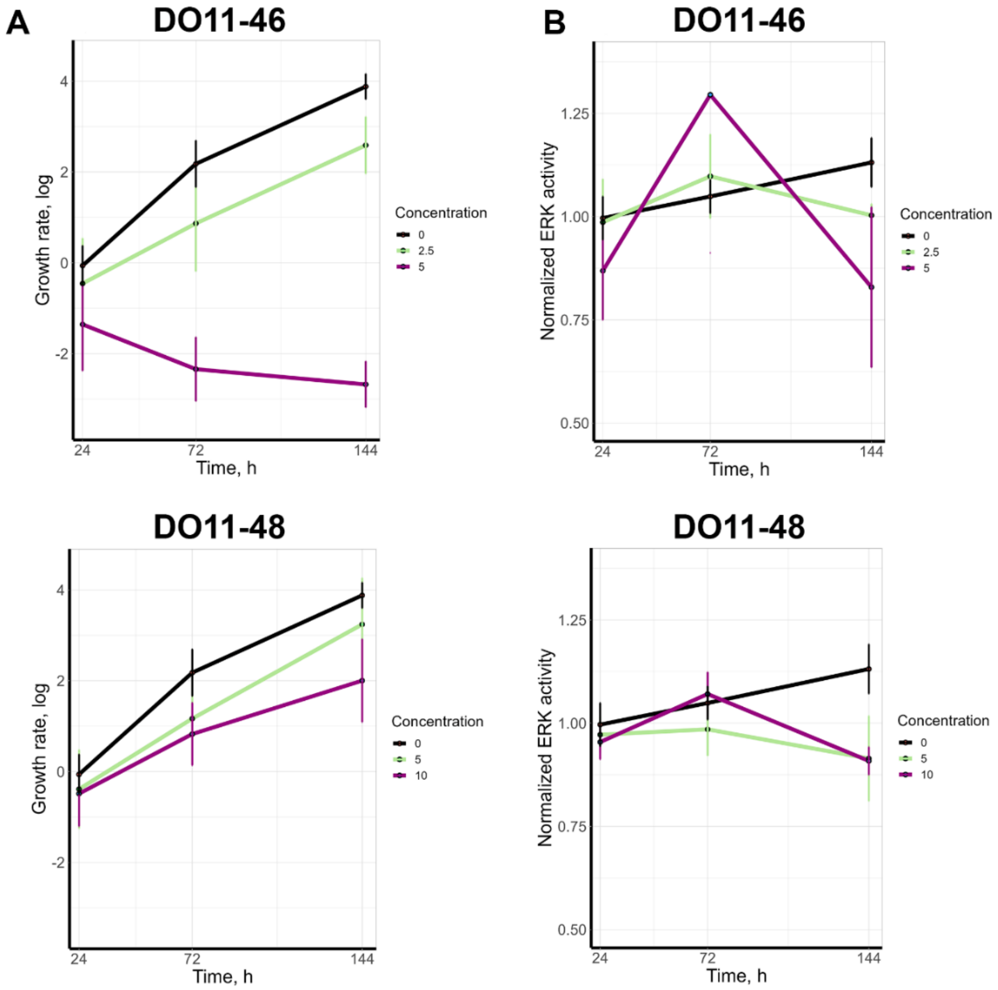

Figure S1. Measurement of ERK1/2 activity and cell proliferation. (A) Changes in cell numbers during treatment with DO11-46 and DO11-48 for 144h. Growth rate was calculated as a log2 ratio of number of cells to the number of initially plated cells. (B) ERK1/2 activity measured by ERK-KTR reporter. ERK activity is presented as a mean ratio of ERK-KTR fluorescence in the cytoplasm to nucleus, and is normalized on ERK activity at 24h in control cells (with drug concentration zero).

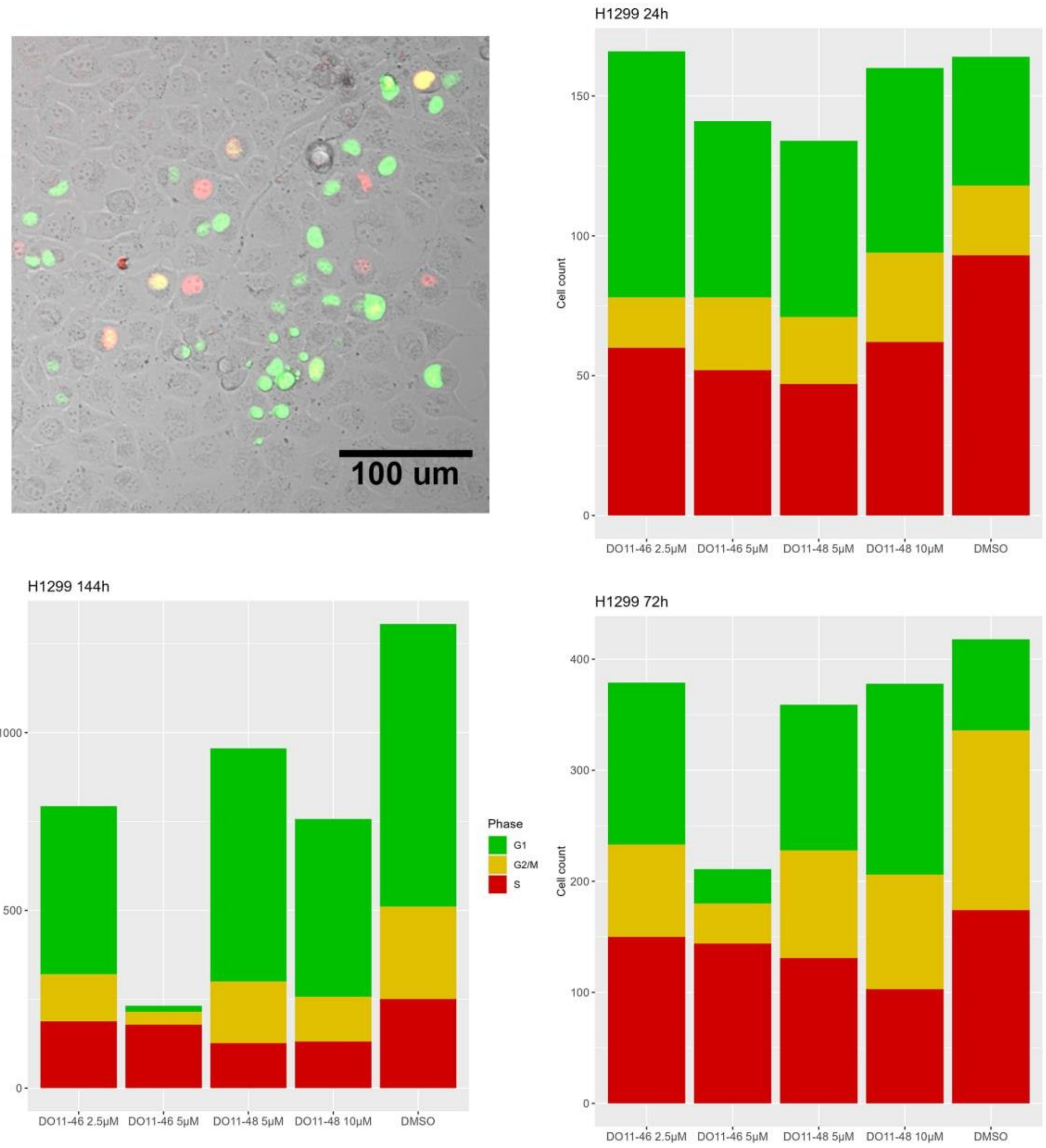

Figure S2. Measurement of cell cycle progression. Representative image of H1299 with PIP-FUCCI reporter. Cells with green nuclei are in G1 phase, with red nuclei in S phase, and with yellow are in G2. The graph shows the number of H1299 cells in the phases of the cell cycle (G1, S, G2/M) treated with DO11-46 (2.5µM and 5µM), DO11-48 (2.5µM and 5µM) or DMSO for 24h, 72h or 144h.
